# The Effect of Artichoke Leaf Extract on Alanine Aminotransferase and Aspartate Aminotransferase in the Patients with Nonalcoholic Steatohepatitis 

**DOI:** 10.1155/2016/4030476

**Published:** 2016-05-11

**Authors:** Vajiheh Rangboo, Mostafa Noroozi, Roza Zavoshy, Seyed Amirmansoor Rezadoost, Asghar Mohammadpoorasl

**Affiliations:** ^1^Department of Nutrition, Faculty of Health, Qazvin University of Medical Sciences, Qazvin, Iran; ^2^Children Growth Research Center, Qazvin University of Medical Sciences, Qazvin, Iran; ^3^Department of Nutrition, Faculty of Nutrition, Qazvin University of Medical Sciences, Qazvin, Iran; ^4^Shahid Chamran Hospital, Shahid Beheshti University of Medical Sciences, Tehran, Iran; ^5^Department of Epidemiology, Tabriz University of Medical Sciences, Tabriz, Iran

## Abstract

*Background*. Based on recent basic and clinical investigations, the extract of artichoke (*Cynara scolymus*) leaf has been revealed to be used for hepatoprotective and cholesterol reducing purposes. We aimed to assess the therapeutic effects of artichoke on biochemical and liver biomarkers in patients with nonalcoholic steatohepatitis (NASH).* Methods*. In a randomized double blind clinical trial, 60 consecutive patients suffering NASH were randomly assigned to receive* Cynara scolymus* extract (as 6 tablets per day consisting of 2700 mg extract of the herb) as the intervention group or placebo as the control group for two months.* Results*. Comparing changes in study markers following interventions showed improvement in liver enzymes. The levels of triglycerides and cholesterol were significantly reduced in the group treated with* Cynara scolymus* when compared to placebo group. To compare the role of* Cynara scolymus* use with placebo in changes in study parameters, multivariate linear regression models were employed indicating higher improvement in liver enzymes and also lipid profile particularly triglycerides and total cholesterol following administration of* Cynara scolymus* in comparison with placebo use.* Conclusion*. This study sheds light on the potential hepatoprotective activity and hypolipidemic effect of* Cynara scolymus* in management of NASH. This clinical trial is registered in the IRCT, Iranian Registry of Clinical Trials, by number IRCT2014070218321N1.

## 1. Introduction

Nonalcoholic fatty liver disease (NAFLD) refers to a wide spectrum of disorders characterized by fatty infiltration in the liver and steatosis [1]. By developing oxidative stress, hepatocellular inflammation, and steatosis, the term was replaced by nonalcoholic steatohepatitis (NASH) that may culminate in cirrhosis and hepatocellular carcinoma [2, 3]. Within the last decade, prevalence of NASH has interestingly doubled especially in the Middle East, Far East, Africa, the Caribbean, and Latin America due to its close association with lifestyle disorders such as diabetes and obesity [4]. In this regard, the best treatment approaches for this phenomenon include weight loss, changes in dietary regimens, and lifestyle modifications. Also, in cases with documented hyperlipidemia or diabetes, use of insulin sensitizing and lipid lowering drugs can be also considered [5]. However, since NASH is a multifactorial disorder, single target based therapy has limited implications. Hence, the use of herbal medicine approach can be a promising alternative due to its multipronged mechanisms of action [6–8].

Artichoke (*Cynara scolymus*) is a plant frequently grown in Mediterranean countries that is rich in natural antioxidants and thus is used as a herbal drug [9]. Based on recent basic and clinical investigations, the extract of artichoke leaf has been revealed to be used for hepatoprotective [10–12], antimicrobial [13], and cholesterol reducing purposes [14].

Artichoke has been found to decrease the production of reactive oxygen species, the oxidation of low-density lipoproteins [15], lipid peroxidation [11], and protein oxidation and increase the activity of glutathione peroxidase [16]. In this regard, it seems that the use of this herb may be promising to treat NASH. The present study aimed to assess the therapeutic effects of artichoke on biochemical and liver biomarkers in patients with NASH.

## 2. Subjects and Methods

### 2.1. Study Population

In a randomized double blind clinical trial, 60 consecutive patients who suffered NASH (based on changes in liver enzymes and sonographic evidences) were included in the study. The main inclusion criteria were elevation of liver enzymes (>30 *μ*/L), any evidences of fatty liver in abdominal sonography, and the existence of at least one of these characteristics: total cholesterol > 200 mg/dL, high density lipoprotein (HDL) < 40 mg/dL for men and <50 mg/dL for women, serum triglycerides level > 150 mg/dL, fasting blood sugar > 100 mg/dL, obesity defined as body mass index > 30 kg/m^2^, or blood pressure > 130/85 mmHg. In this regard, the main exclusion criteria were daily alcohol consumption, diabetes mellitus type I, the existence of concomitant liver diseases such as hepatitis B or C, autoimmune hepatitis, Wilson, hemochromatosis, alpha-1 antitrypsin deficiency, or biliary obstruction, the use of vitamin C, livergol, hepatotoxic drugs, NASH inducing drugs such as amiodarone, calcium-channel blockers, or tamoxifen, pregnancy or breastfeeding, sensitivity to artichoke species, or any life-threatening disorders.

The two groups were matched in terms of sex distribution (21 male and 9 female in both groups) and mean age (47.27 ± 8.12 years in intervention group and 49.83 ± 12.79 years in placebo group, *P* = 0.357). The two groups were also similar in other baseline characteristics including weight, levels of liver enzymes, lipid profile, and fasting blood sugar on initial assessment.

### 2.2. Study Intervention

The baseline characteristics of patients were collected by interviewing and the study questionnaires were recorded. The patients were then randomly assigned to receive* Cynara scolymus* extract (as 6 tablets per day consisting of 2700 mg extract of the herb prepared in Dineh company, Qazvin, Iran) as the intervention group or placebo as the control group for two months (placebo was prepared from the same ingredient as treatment except* Cynara scolymus* extract). The randomization was done using computer generated random number tables. Both groups were advised to maintain regular physical activity (20 min walking within 5 days a week) and an appropriate dietary regimen (calculated based on patients' weight, height, age, and percentage of activity using the Mifflin formula to gradually reduce body weight).

### 2.3. Measurement of Biomarkers

Serum alanine transaminase (ALT) and aspartate transaminase (AST) activity was estimated colorimetrically using an especial kit (Pars Azmoon company, Iran) according to the method of Reitman and Frankel [17]. Serum cholesterol (Chol) concentration was determined colorimetrically using an especial kit (Pars Man company, Iran) according to the method of Allain et al. [18]. Serum LDL-cholesterol (LDL) concentration was assayed colorimetrically using an especial kit (Pars Azmoon company, Iran) according to the method of Assmann et al. [19]. Serum HDL-cholesterol (HDL) concentration was measured colorimetrically using an especial kit (Pars Man company, Iran) according to the method of Lopez-Virella et al. [20]. Serum triglycerides (TG) level was determined colorimetrically using an especial kit (Pars Azmoon company, Iran) according to the method of Fassati and Prencipe [21]. The biomarkers were measured at the two time points: admission time and 2 months after the initial assessment (completing treatment protocols).

### 2.4. Statistical Analysis

Results were presented as mean ± standard deviation (SD). Continuous variables were compared using *t*-test or nonparametric Mann-Whitney *U* test. The changes in study biomarkers after interventions were assessed using the Paired *t*-test or nonparametric Wilcoxon test. The multivariate linear regression analysis was used for assessing study outcomes following different employed protocols as follows. First, the baseline variables as probable confounders with a *P* value <0.20 in univariate analyses were taken in a multivariate logistic regression model to assess the difference between the treatment groups receiving* Cynara scolymus* extract or placebo with the presence of these confounders. For the statistical analysis, the statistical software SPSS version 21.0 for windows (SPSS Inc., Chicago, IL) was used.

## 3. Results

As shown in Table 1, following administration of* Cynara scolymus*, significant reduction was observed in mean weight, as well as in serum levels of ALT, AST, blood sugar, total cholesterol, LDL, and triglycerides. Also, mean systolic blood pressure was significantly reduced using* Cynara scolymus*. In contrast, using placebo resulted in significant reduction in mean weight, as well as in serum levels of ALT and AST; however, among other biomarkers, blood sugar and lipid profile remained unchanged. Comparing changes in study markers following interventions showed the changes in liver enzymes, and also levels of triglycerides and cholesterol were significantly more in group treated with* Cynara scolymus* when compared to placebo group (Table 2). To compare the role of* Cynara scolymus* use with placebo in changes in study parameters, multivariate linear regression models were employed (Table 2) indicating higher improvement in liver enzymes and also lipid profiles of triglycerides and total cholesterol following administration of* Cynara scolymus* in comparison with placebo use.

## 4. Discussion

Our study aimed to assess the beneficial effects of* Cynara scolymus,* a herb with antioxidant compounds, on liver function and also lipid profile and fasting blood glucose. On the other hand, we aimed to demonstrate therapeutic effects of this herb on liver functional biomarkers and also hemodynamic parameters in patients with NASH. In this double blind clinical trial, patients in intervention group received* Cynara scolymus* extract for two months and changes in study biomarkers were reassessed.

Serum ALT and AST are effective biomarkers in the diagnosis of hepatic damage. Sever liver damage was demonstrated by remarkable elevation of serum ALT and AST levels. This elevation may be attributed to the release of these enzymes from the cytoplasm in to the blood circulation after rupture of the plasma membrane and cellular damage [22].

The result of the present study showed significant changes in serum ALT and AST levels in intervention group in comparison with the placebo group (*P* < 0.001). This effect could be attributed to the antioxidant ingredients in* Cynara scolymus* extract such as mono- and dicaffeoylquinic acid (cynarin and chlorogenic acid), caffeic acid and flavonoids including the glycosides luteolin-7-*β*-rutinoside (scolymoside), luteolin-7-*β*-D-glucoside, and luteolin-4-*β*-D-glucoside that are mainly compounds in* Cynara scolymus* extract [23–25]. And chlorogenic acid is the most active antioxidant in* Cynara scolymus* extract [26]. Antioxidants are components that prevent oxidative reactions often by scavenging free radicals before they can damage cells [27, 28]. Several in vitro and animal studies assessed the antioxidative and free radical scavenging potential of artichoke extracts in protection hepatocytes from oxidative stress [29–32], but there are a few human and clinical studies in this regard. The result of the present study in reduction of serum ALT and AST levels is in agreement with Saffa et al. They investigate the efficacy of* Cynara scolymus *total methanolic extract (CSM) and its fraction (CSF) in rat. Their result showed that CSF is more active in comparison with CSM. Perhaps it is because of high concentration of monocaffeoylquinic acid derivatives (chlorogenic acid) in CSF [33]. In Huber's study although the used doses of* Cynara scolymus* extract and duration of study were more than the present study, remarkable reduction in serum ALT and AST levels was not seen in patients with hepatitis C, which is not in agreement with our study. It appears that because of microbial agent and sever damage of hepatocytes uneffectiveness of* Cynara scolymus *extract on ALT and AST was caused [34]. Also, in present study, improvement of lipid profile was significantly observed in the group receiving* Cynara scolymus* extract not in placebo group. These effects could be attributed to active ingredients in* Cynara scolymus* extract which are known as caffeoylquinic acid derivatives (cynarin and chlorogenic acid). Some previous studies showed that these compounds can reduce cholesterol by inhibiting HMG-COA reductase and having a hypolipidemic influence lowering blood cholesterol [26].

Our result on hypocholesterolemic effect of* Cynara scolymus* extract is in agreement with Pittler et al. [35]. Some studies suggest that* Cynara scolymus* extract reduces blood lipids by directly influencing biosynthesis of cholesterol and also by production and secretion of bile from the liver [36, 37]. Reduction in triglycerides level is attributed to improvement in glycemic control and reduction of glucose instead of fat. Acetyl COA yield from pyruvic acid enters Krebs cycle and leads to metabolism of glucose completely instead of triglycerides biosynthesis.

In present study improvement in fasting blood sugar was significantly observed in group receiving* Cynara scolymus* extract not in placebo group. This beneficial effect could be attributed to high antioxidant capacity of this herb. Phenolic compounds, specially, such as caffeic acid and flavonoids, are representative of this effect.

Another mechanism for reducing glucose level by* Cynara scolymus* extract is affecting glucose absorption. Antioxidant compounds delay depletion of stomach and bowels and they inhibited *α*-amylase and *α*-glucosidase enzymes in bowels and blocked glucose transportation to blood. On the other hand antioxidants have insulin-like effect and increase glucose absorption in peripheral tissue. Another probable mechanism is influencing *β*-cells, repairing damage cells, and stimulating these cells to secrete insulin. Studies show that chlorogenic acid has antidiabetic effect [38, 39] and reduction glucose by reason of this compound.

Another point of this study was the parallel improvement in liver enzymes and also lipid profile by administrating* Cynara scolymus* that was not simultaneously in placebo group. Cholesterol metabolism is associated with liver fat content independent of body weight, implying that the more the fat the liver contains, the higher the cholesterol synthesis is [40]. Cellular cholesterol synthesis is regulated by activation of membrane bound transcription factors, designated sterol regulatory element-binding proteins (SREBPs) which are the most abundant in the liver [41], and the excess of cellular cholesterol is esterified by the acyl CoA-cholesterol acyltransferase (ACAT) [42]. The high level of cholesterol synthesis and the increased SREBP-2 activity have paradoxically been shown in subjects with NASH [43]. It is thus suggested that the effects of* Cynara scolymus* may appear by inducing and involving these metabolic pathways in the liver.

The main strength of the study was to shed light on the potential role of artichoke on biochemical and liver biomarkers in patients with NASH in a controlled trial. Although, in previous studies, the role of this herb to treat NASH has been assessed, a few studies focused directly on the improvement of liver enzymes* via* administrating this herbal drug. Another strength of the current study was to assess the simultaneous changes in liver enzymes, blood sugar, and lipid profile. This concomitant assessment is important because the central role of liver leads to metabolic pathways regulating the level of these metabolic biomarkers. However, our study had some potential limitations including small sample size leading to partially low study power as well as ignoring other baseline clinical and pharmacological confounders affecting the employed regression models used for assessing the effects of artichoke on biochemical and liver biomarkers in NASH.

## 5. Conclusion

In conclusion, the current study sheds light on the potential role of* Cynara scolymus* in management of NASH. The active constituents of this herb such as flavonoids and caffeoylquinic acid may be responsible for this effect. These compounds have been proven to have hepatoprotective activity and hypolipidemic effect.

## Figures and Tables

**Table 1:** Changes in study biomarkers in intervention and placebo groups.

Marker	Intervention group			Placebo group			Intergroup difference
	Before	After	*P* value	Before	After	*P* value	
Weight (kg)	83.90 ± 15.83	79.28 ± 14.25	<0.001	81.81 ± 14.25	77.05 ± 14.83	<0.001	0.859
ALT (mg/dL)	81.77 ± 38.73	38.40 ± 14.15	<0.001	74.13 ± 23.61	64.07 ± 20.36	<0.001	<0.001
AST (mg/dL)	45.53 ± 13.78	24.60 ± 7.43	<0.001	44.50 ± 9.82	39.60 ± 10.41	<0.001	<0.001
FBS (mg/dL)	108.07 ± 28.90	97.60 ± 14.50	0.029	107.43 ± 18.48	102.57 ± 10.78	0.096	0.302
Chol (mg/dL)	206.47 ± 31.20	182.87 ± 34.64	0.001	213.67 ± 48.48	211.63 ± 48.96	0.686	0.008
LDL (mg/dL)	122.14 ± 30.42	108.12 ± 32.36	0.039	116.09 ± 29.38	113.23 ± 31.79	0.659	0.120
HDL (mg/dL)	45.87 ± 10.46	43.33 ± 8.20	0.129	45.50 ± 7.50	44.87 ± 6.10	0.689	0.403
TG (mg/dL)	193.37 ± 86.03	154.50 ± 84.93	0.011	179.87 ± 45.67	184.23 ± 57.83	0.659	0.016
SBP (mg/dL)	132.70 ± 13.55	126.57 ± 8.54	0.004	130.23 ± 10.34	128.20 ± 6.62	0.044	0.070
DBP (mg/dL)	80.00 ± 9.21	79.03 ± 6.89	0.578	80.87 ± 8.52	81.63 ± 7.31	0.504	0.403

**Table 2:** Multivariate linear regression models for determining the role of *Cynara scolymus* use on changes in liver enzymes and lipid profile.

Marker	Variable	Beta	95% confidence interval	*P* value
ALT change	Artichoke use	−31.365	−45.201 to −17.528	<0.001
	Male sex	−0.634	−16.252 to 14.983	0.935
	Age	−0.754	−1.434 to −0.074	0.030

AST change	Artichoke use	−16.041	−21.405 to −10.677	<0.001
	Male sex	−2.995	−9.049 to 3.060	0.326
	Age	0.003	−0.261 to 0.266	0.983

Chol change	Artichoke use	−21.673	−37.245 to −6.101	0.007
	Male sex	−17.072	−34.648 to 0.505	0.057
	Age	0.041	−0.723 to 0.806	0.914

TG change	Artichoke use	−41.960	−76.613 to −7.307	0.019
	Male sex	−28.315	−67.428 to 10.798	0.153
	Age	−0.496	−2.198 to 1.206	0.562
